# Long-Term Spinal Ventral Root Reimplantation, but not Bone Marrow Mononuclear Cell Treatment, Positively Influences Ultrastructural Synapse Recovery and Motor Axonal Regrowth

**DOI:** 10.3390/ijms151119535

**Published:** 2014-10-28

**Authors:** Roberta Barbizan, Mateus V. Castro, Rui Seabra Ferreira Jr., Benedito Barraviera, Alexandre L. R. Oliveira

**Affiliations:** 1 Department of Structural and Functional Biology, University of Campinas (UNICAMP), PO Box 6109, Campinas 13083-970, São Paulo, Brazil; E-Mails: robertabarbizan@yahoo.com.br (R.B.); mateusvidigal@hotmail.com (M.V.C.); 2 Center for the Study of Venoms and Venomous Animals (CEVAP), São Paulo State University (UNESP), Botucatu 18610-307, São Paulo, Brazil; E-Mails: rui.ead@gmail.com (R.S.F.J.); bbviera@gnosis.com.br (B.B.)

**Keywords:** ventral root avulsion, fibrin sealant, root reimplantation, mononuclear cells, ultrastructure

## Abstract

We recently proposed a new surgical approach to treat ventral root avulsion, resulting in motoneuron protection. The present work combined such a surgical approach with bone marrow mononuclear cells (MC) therapy. Therefore, MC were added to the site of reimplantation. Female Lewis rats (seven weeks old) were subjected to unilateral ventral root avulsion (VRA) at L4, L5 and L6 levels and divided into the following groups (*n* = 5 for each group): Avulsion, sealant reimplanted roots and sealant reimplanted roots plus MC. After four weeks and 12 weeks post-surgery, the lumbar intumescences were processed by transmission electron microscopy, to analyze synaptic inputs to the repaired α motoneurons. Also, the ipsi and contralateral sciatic nerves were processed for axon counting and morphometry. The ultrastructural results indicated a significant preservation of inhibitory pre-synaptic boutons in the groups repaired with sealant alone and associated with MC therapy. Moreover, the average number of axons was higher in treated groups when compared to avulsion only. Complementary to the fiber counting, the morphometric analysis of axonal diameter and “g” ratio demonstrated that root reimplantation improved the motor component recovery. In conclusion, the data herein demonstrate that root reimplantation at the lesion site may be considered a therapeutic approach, following proximal lesions in the interface of central nervous system (CNS) and peripheral nervous system (PNS), and that MC therapy does not further improve the regenerative recovery, up to 12 weeks post lesion.

## 1. Introduction

Brachial plexus injury is a devastating injury, causing significant loss of upper limb function and ability to perform everyday tasks [1]. Usually, such lesion occurs in high-energy trauma, where the neck is stretched from the trunk as in motorcycle accidents, extreme sports as well as severe traction during complicated deliveries [2]. The surgery to repair this injury is very complicated and still has poor clinical results. Animal models were developed to study the brachial plexus injury at cellular level, leading to the improvement and proposal of new strategies for root reimplantation.

Ventral root avulsion (VRA) produces abrupt rupture of the spinal roots from the surface of the spinal cord. It results in disconnection between the neuronal cell body and target muscular fibers with interruption of anterograde flow of neurotrophic factors. Such disconnection generates massive loss of motoneurons [3,4] with functional remodeling of the spinal cord circuitry. Synaptic loss and retraction, which is more pronounced in the neuronal body, can become permanent if reinnervation does not occur [5].

The surgical implantation of the injured rootlets, although a complicated task, can serve as a model for studying regeneration of motor axons from the CNS, towards the PNS [6]. The reimplantation of ventral roots is neuroprotective by itself, and has been shown to allow axonal regeneration and result in a functional and anatomical reinnervation of muscles and urinary tract [7,8,9]. Hallin and colleagues [10] observed greater survival of spinal motoneurons after reimplantation of fourth and fifth cervical ventral roots (C4–C5) in monkeys, suggesting that root repair increases production of neurotrophic factors. In addition, the persistent break of the blood-brain barrier allows trophic substances to reach injured motoneurons [11,12,13].

Bone marrow mononuclear cells (MC) therapy can even enhance the levels of some trophic substances, potentially increasing neuronal survival and functional recovery. It can support nerve regeneration [14] towards the denervated target muscle. This work investigated if root reimplantation, combined with MC transplantation, further improves long-term nerve regeneration and synaptic recovery.

## 2. Results

### 2.1. Ventral Root Avulsion (VRA) Implantation Preserved Synaptic Inputs by Ultrastructural Analysis of Spinal Cord

**Figure 1:**
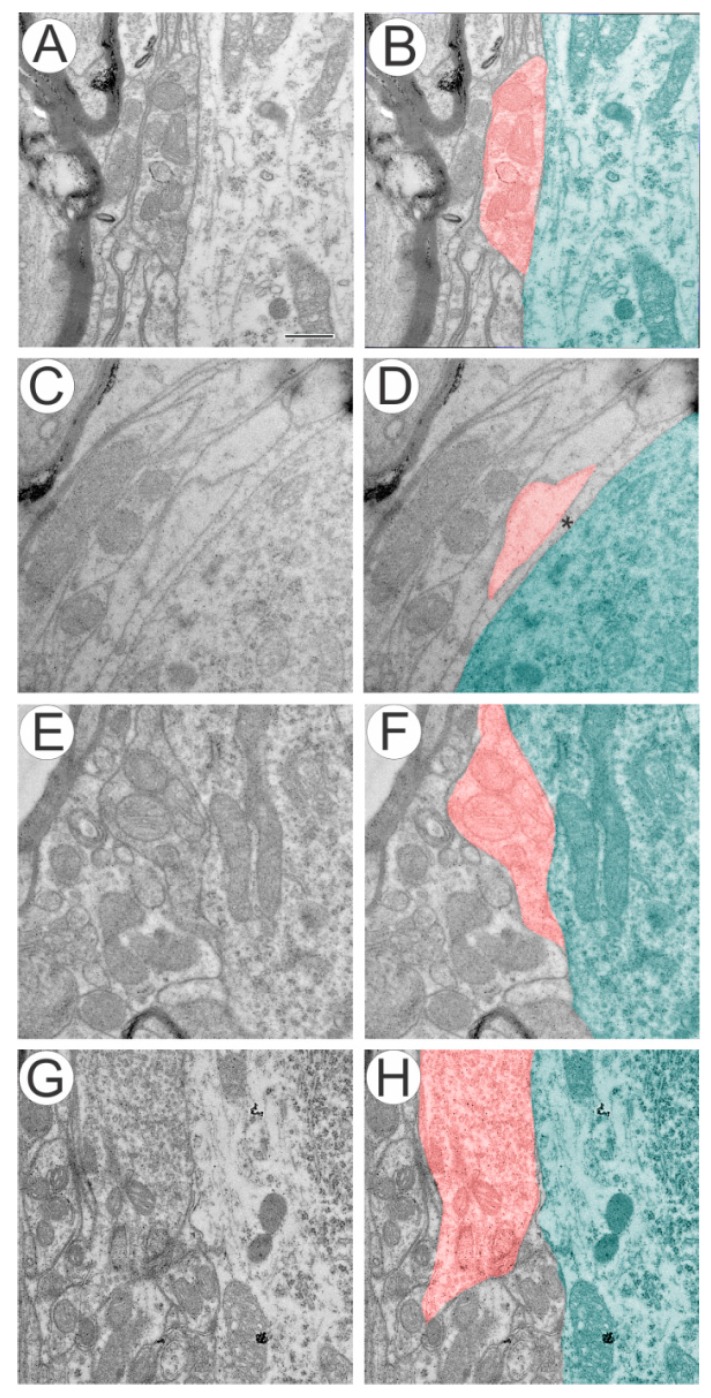
(**A**,**B**) Ultrastructure of synapses apposed to α motoneurons showing normal input apposition on the surface of an α motoneuron; (**C**,**D**) retracted terminal intermingled with an astrocyte projection following ventral root avulsion (*, glial projection); In avulsion + sealant (**E**,**F**) and avulsion + sealant + cell (**G**,**H**) we observed a close to normal synaptic apposition. In **B**, **D**, **F** and **H**, synaptic terminals are highlighted in red and the motoneuron cytoplasm is colored in green. Scale bar = 500 nm.

The ultrastructural analysis of the control side showed extensive covering of the motoneurons synaptic membrane (Figure 1A,B). In the avulsed group, distinct ultrastructural changes around the membrane of motoneurons could be identified (Figure 1C,D), and a number of presynaptic terminals were retracted. Glial projections were also commonly observed in contact with the postsynaptic membrane (Figure 1D), filling the space between synaptic terminals and post-synaptic membrane. Reimplanted groups, with or without MC treatment, presented close to normal synaptic covering (Figure 1E–H).

**Figure 2:**
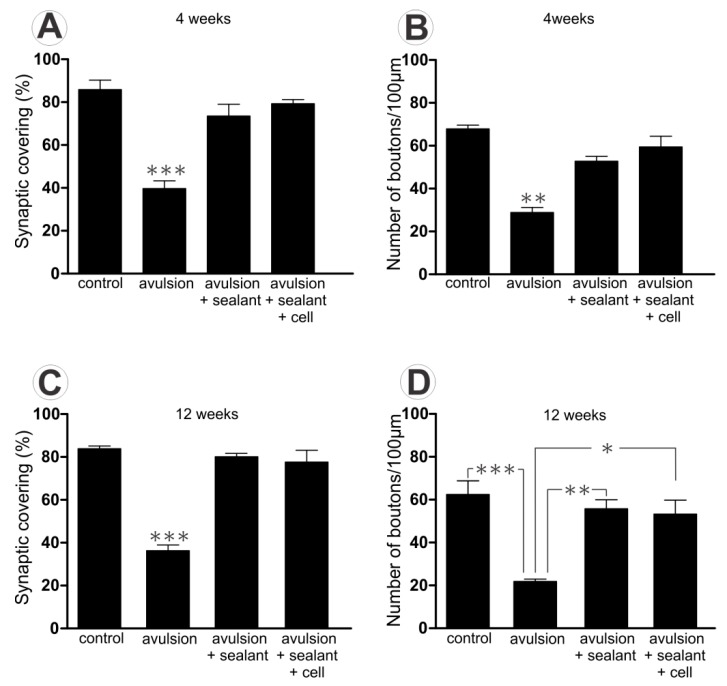
Quantitative ultrastructural analysis of synaptic covering (**A**) and normalized number of boutons per 100 µm of motoneuron membrane (**B**) 4 weeks after lesion; (**C**) and (**D**) represent the same results after 12 weeks post injury. Observe a reduction in synaptic covering following avulsion alone, 4 and 12 weeks after avulsion. (* *p* < 0.05; ** *p* < 0.01; *** *p* < 0.001).

The analysis of the percentage of synaptic covering and the number of terminals showed reduced synaptic contact to motoneuron cell body (Figure 2) in the avulsed group compared to the other examined groups, both with 4 or 12 weeks. However, there were no differences among the same group over time. (Percentage of synaptic inputs 4 weeks control: 85.84 ± 4.45; avulsion: 39.72 ± 3.53; avulsion + sealant: 73.51 ± 5.50; avulsion + sealant + cells: 79.33 ± 1.84 and 12 weeks control: 84.82 ± 1.32; avulsion: 37.67 ± 2.511; avulsion + sealant: 79.09 ± 2.44; avulsion + sealant + cells: 77.96 ± 5.50. The normalized number of inputs per 100 µm of membrane yield similar results (4 weeks control: 67.89 ± 1.70; avulsion: 28.86 ± 2.35; avulsion + sealant: 52.77 ± 2.26; avulsion + sealant + cells: 59.46 ± 4.98 and 12 weeks control: 61.14 ± 5.08; avulsion: 23.47 ± 1.85; avulsion + sealant: 55.03 ± 3.31; avulsion + sealant + cells: 57.53 ± 6.31).

#### 2.1.1. Preferential Loss of Excitatory Terminals after Avulsion

**Figure 3:**
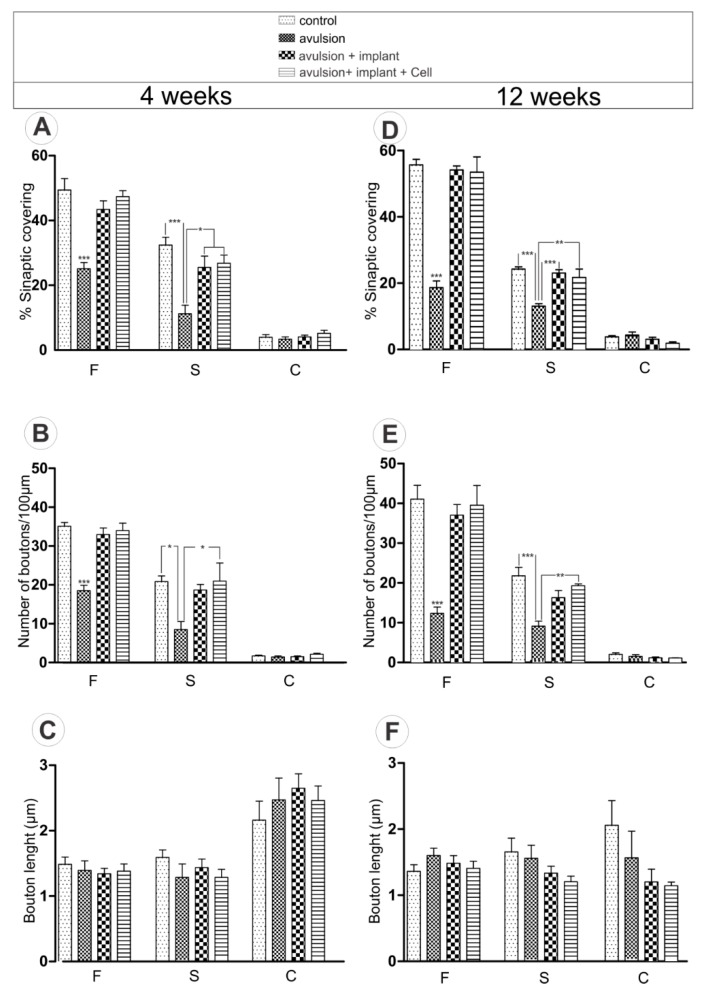
Detailed quantitative analysis of *F*-, *S-* and *C*-terminals. (**A**) Synaptic covering, (**B**) Number of boutons/100 µm of motoneuron membrane and (**C**) Bouton length at 4 weeks after injury; (**D**) Synaptic covering, (**E**) Number of boutons/100 µm of motoneuron membrane and (**F**) Bouton length at 12 weeks after injury. (* *p* < 0.05; ** *p* < 0.01; *** *p* < 0.001).

Specific synaptic bouton typing in apposition to the cell body was also carried out. The *F*, *S*, and *C*-terminals, representing the inhibitory (GABA and/or glycine), excitatory (glutamatergic), and cholinergic inputs, respectively, were analyzed. (Figure 3). In this regard, a more prominent loss of excitatory inputs (*S*-terminals) was observed after VRA 4 weeks following avulsion, indicating an imbalance between inhibitory and excitatory synapses. Avulsion group presented 65.36% less covering of *S*-terminals, compared to control, 4 weeks after surgery. On the other hand, avulsion + sealant decreased 21.22% and avulsion + sealant + cell, 17.30%, indicating preservation of spinal circuits. Additionally, the total loss of *F*-terminals, 4 weeks after lesion, was 49.21%, 12.19% and 4.20% in avulsion, avulsion + sealant and avulsion + sealant + cells, respectively. Similar results were obtained following 12 weeks after injury (*S*-terminals—Avulsion 45.91% less covering of *S*-terminals as compared to 12 weeks after surgery control, avulsion + sealant 4.84% and avulsion + sealant + cell 10.30%). *F*-terminal loss after 12 weeks was 66.35%, 2.72% and 3.99% in avulsion, avulsion + sealant and avulsion + sealant + cells, respectively.

#### 2.1.2. Pattern of the Terminal Distribution after Avulsion

**Figure 4:**
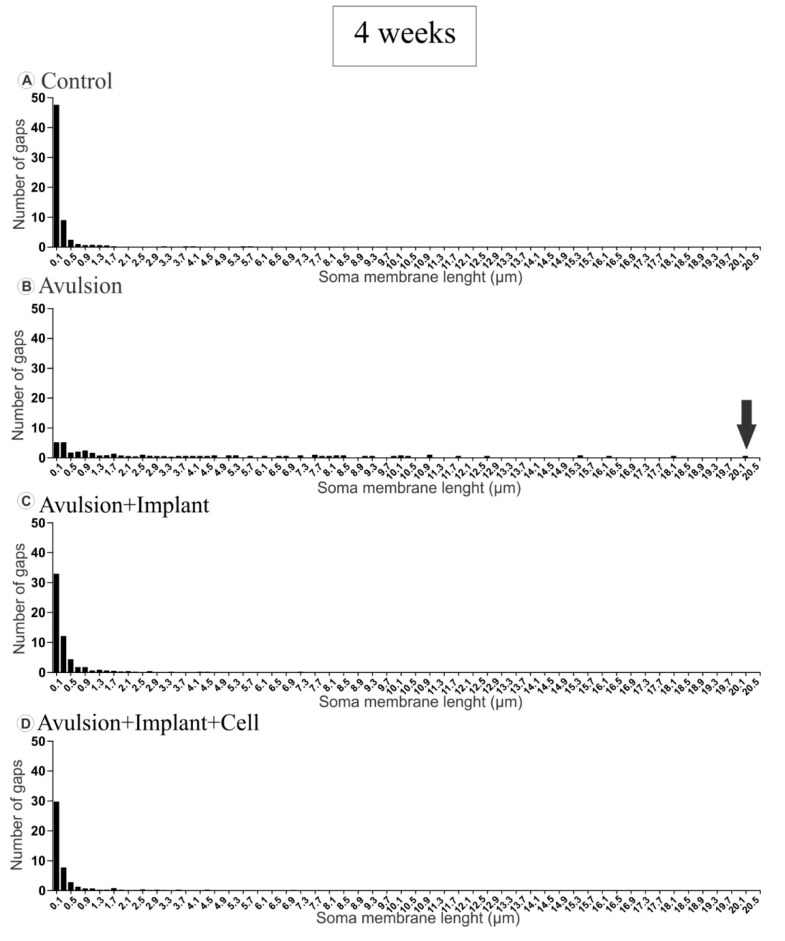
Distribution of gap size between clusters of terminals apposing the motoneuron membrane, 4 weeks after injury. (**A**) Normal distribution of intervals between nerve terminals showing that boutons are organized in clusters of inputs; (**B**) Distribution of gaps in avulsed animals showing long intervals between boutons (arrow showing the largest gap found, in comparison to the other groups), as a result of extensive input loss; (**C** and **D**) Reduction of gap size between terminals after root reimplantation (**C**) and reimplantation combined with mononuclear cell therapy (**D**).

**Figure 5:**
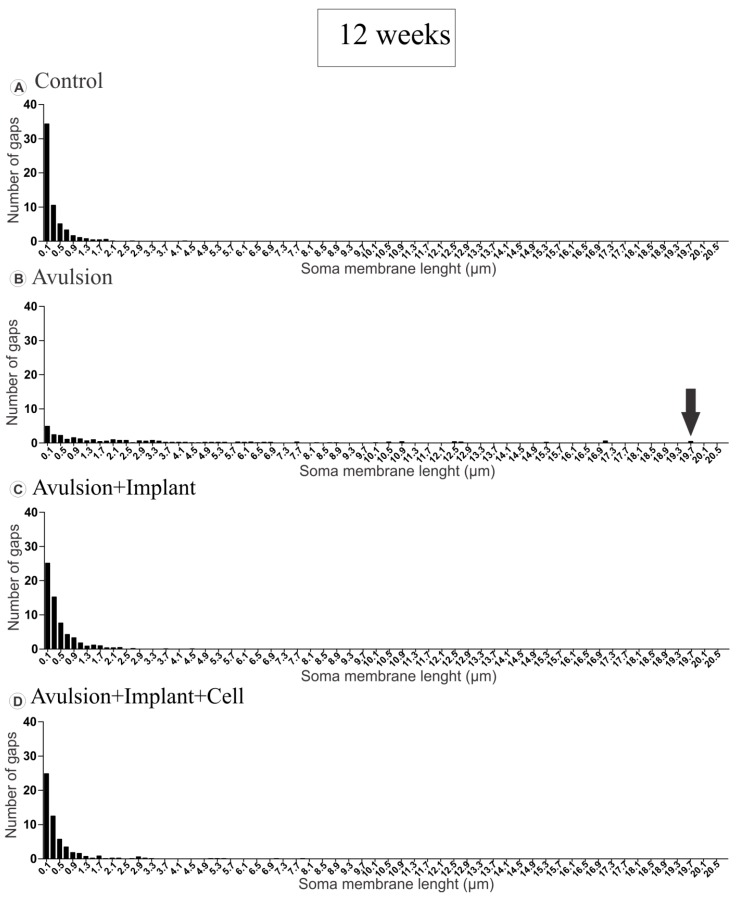
Distribution of gap length between clusters of terminals apposing to motoneuron membrane, 12 weeks after avulsion. (**A**) Normal distribution of intervals between nerve terminals showing that boutons are organized in clusters of inputs; (**B**) Distribution of gaps in avulsed animals showing long intervals between boutons (arrow showing the largest gap found, in comparison to the other groups), as a result of significant input loss; (**C** and **D**) Reduction of gap size between terminals after root reimplantation (**C**) and reimplantation combined with mononuclear cell therapy (**D**).

In normal neurons, the terminals presented in clusters, with few intervals between inputs. However, in the avulsed group, the gaps between clusters of boutons tended to increase after retraction of inputs, which reached up to 20.1 µm after 4 weeks (Figure 4B) and 19.7 µm at 12 weeks post lesion (Figure 5B). The reimplanted groups showed an intermediate pattern between the non lesioned and the avulsed groups with, however, greater similarity to the control (Figure 4 and Figure 5).

### 2.2. VRA Implantation Promotes Axonal Regeneration Observed by Histological Analysis of the Sciatic Nerve

**Figure 6:**
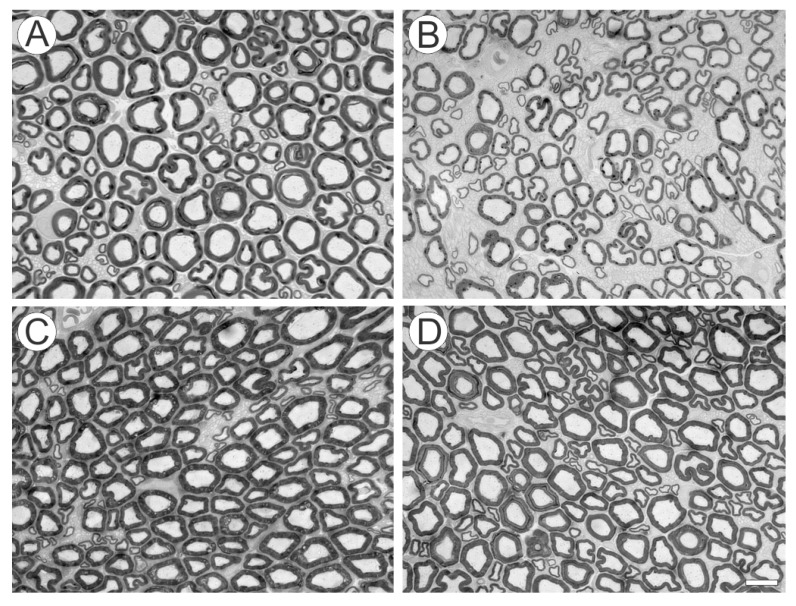
Transverse cross sections of sciatic nerves, 12 weeks after injury. Observe the decreased number of myelinated fibers in the avulsion group as compared to reimplanted groups. (**A**) control; (**B**) avulsion; (**C**) avulsion + sealant; (**D**) avulsion + sealant + cells. Scale bar = 10 µm.

**Figure 7:**
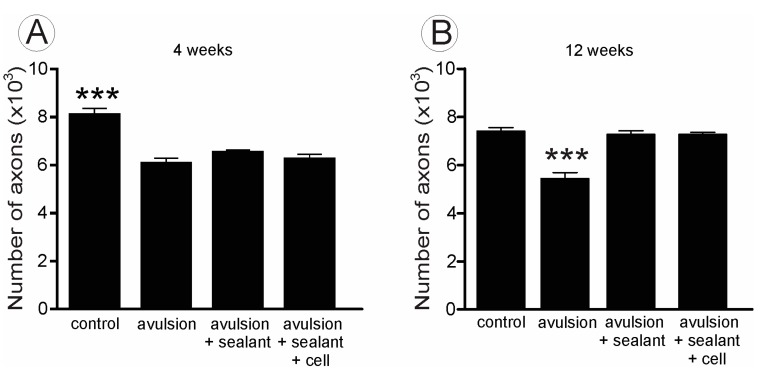
Number of myelinated fibers estimated for the sciatic nerve, 4 (**A**) and 12 (**B**) weeks after injury. The avulsion group presents fewer myelinated fibers compared with the other groups. (*** *p* < 0.001).

**Figure 8:**
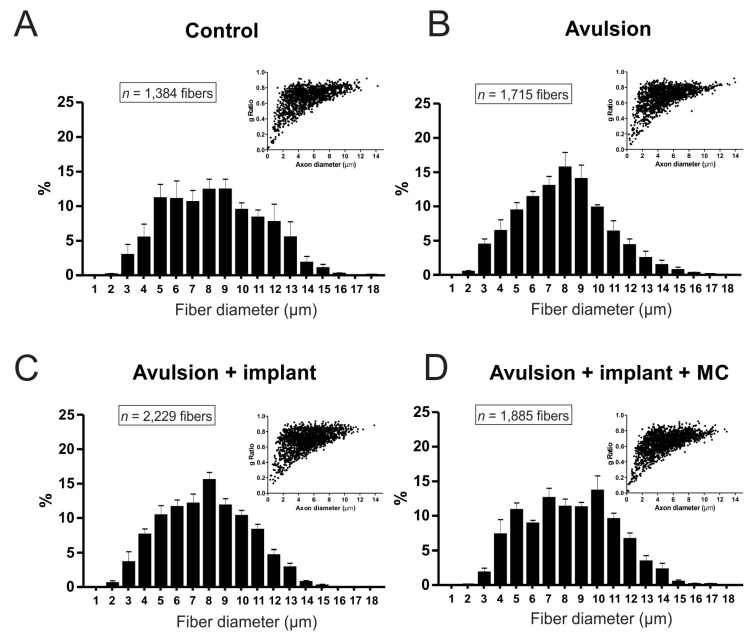
Morphometry in the different experimental groups, 12 weeks post injury (**A–D**) Frequency distribution of fiber diameter and Scatter plots of “g” ratio *versus* axon diameter (inset). Observe avulsion only presents a reduction of large axons that is partially recovered especially after root reimplantation combined with mononuclear cells. Nevertheless, the correlation of axon size and myelin thickness is not changed as a whole, since only L4–L6 motor axons were lesioned.

In the sciatic injured nerves, at 4 weeks after root reimplantation, the number of fibers was reduced in all experimental groups in comparison to the contralateral intact nerves (Figure 6 and Figure 7A). No statistical differences were observed between groups at this stage. On the other hand, at 12 weeks after root reimplantation, the number of axons increased in both treated groups, when compared to VRA without reimplantation (Figure 7B), confirming the capacity of regeneration of motor axons after reimplantation, allowing growth up to the target muscles. In addition, morphometry revealed fibers with smaller caliber and “g” ratio in in the sciatic nerve of the animals with VRA without reimplantation, when compared with the treated groups and to the contralateral nerve (Figure 8, Table 1). Contralateral intact sciatic nerves showed normal distribution and greater number of fibers.

**Table 1:** Mean values of myelinated fiber diameter and “g” ratio in the different experimental groups 12 weeks post lesion. Values are presented as mean ± SE.

Groups	Fiber Diameter (µm)	“g” Ratio
**Control**	7.59 ± 0.40	0.66 ± 0.03
**Avulsion**	7.32 ± 0.23	0.69 ± 0.02
**Avulsion + Implant**	7.25 ± 0.14	0.65 ± 0.02
**Avulsion + Implant + MC**	7.73 ± 0.28	0.62 ± 0.02

## 3. Discussion

Ventral root avulsion (VRA) characterizes by the abrupt disconnection of spinal rootlets from the spinal cord surface, resulting in immediate limb paralysis [15]. Such proximal mechanical lesion results in disconnection between the neuronal body and its target muscle fibers. Associated with this, there is an acute interruption of the anterograde flow of neurotrophic factors produced by the target organ.

VRA lesion is increasingly frequent in humans, associated with brachial plexus injury following high-energy accidents. To date, repair approaches for such lesion give limited results, which reinforces the need for improvement by developing alternative treatments. In the present work, we performed ventral root reimplantation at the avulsion site, concomitantly with MC engrafting. The results show that the proposed experimental approach enhances the number of regenerating fibers, also increasing synaptic stability at the spinal cord up to 12 weeks after reimplantation. However, no significant differences, in relation to the reimplantation alone, could be depicted in the long-term analysis. This, however, does not necessarily mean that cell therapy is not effective. In fact, we have previously shown that a similar therapeutic approach, following ventral root crushing, yields positive results up to the fourth week following lesion. In this sense, a significant preservation of spinal cord circuits was observed, which may in turn contribute to a more consistent sensory-motor recovery at later survival times [16]. One possibility is that MC produce neurotrophins that contribute to motoneuron survival after nerve root lesion [15,16]. This may probably occur in the first or second week post engraftment, not persisting in the long term, since a significant amount of MC die by either apoptosis or necrosis. Nevertheless, a number of surviving MC can be found at the lesion site even after 12 weeks post lesion, indicating that repetition of cell therapy may extend the neurotrophic production window. It is important to highlight that Li *et al.* studied the beneficial effects of olfactory ensheathing cells following ventral root avulsion and reimplantation [17]. In this sense, S1 ventral root was avulsed and reimplanted with the use of Tisseal fibrin glue, with the opening of a pial pocket. The results were observed by immunohistochemistry, in a semiquantitative fashion, indicating a significant enhancement of root axonal regrowth, two weeks after surgery. Such results are in line with a recent work from our lab, using mononuclear cells from the bone marrow [18]. Nevertheless, we have shown that the acute positive results of cell therapy do not last for more than four weeks, indicating that additional cell administration may be necessary to sustain the protective results.

A hallmark of ventral root avulsion is the synaptic stripping process that takes place in the motoneuron cell body and proximal dendrites [5,6,8,19,20,21,22,23]. Such a process decreases or even abolishes synaptic transmission [24,25,26]. In the case of reinnervation to the target organ not occurring, such synaptic changes may become irreversible [5]. In agreement with the literature, we observed extensive synapse loss after avulsion without reimplantation. Root reimplantation at the site of avulsion alone already produced significant stabilization of such inputs. No significant difference, however, could be observed with the MC therapy, reinforcing the idea that stem cell therapy may have to be repeated in order to keep the positive effects observed in the short term.

An important finding, regarding the arrangement of the pre-synaptic terminals apposed to the surface of axotomized motoneurons is that reimplantation preserved clusters of boutons. This contrasts with the avulsion without reimplantation, which results in large gaps between the remaining terminals, possibly making them less efficient in terms of cell depolarization. Such gaps are occupied with glial processes, which is in line with the enhanced astrogliosis in the avulsion alone group, 12 weeks after injury, in comparison then in the reimplanted counterpart [27].

Besides assessing the synaptic elimination process between groups, we also typed the terminals in apposition to the motoneuron membrane. As expected, a preferential loss of excitatory inputs over inhibitory boutons is depicted, so that axotomized motoneurons reached a resting state in terms of synaptic transmission [28]. This stereotyped reaction is possibly related to the avoidance of excitotoxic effects of glutamatergic inputs, that may in turn lead to cell death via apoptosis or necrosis [28,29]. This has been reported before following proximal axotomy of motor axons at the ventral funiculus of the spinal cord [28]. Such selectivity for glutamatergic terminals is probably controlled by the motoneuron itself as the result of a metabolic shift from a state of synaptic transmission to a state of recovery.

The fibrin sealant root reimplantation at the avulsion site seems to be essential for the axon regrowth, exiting the CNS in order to reach the target muscle fibers. The success of axonal regeneration following root repair becomes clear by analyzing the sciatic nerve, 12 weeks post-surgery. Avulsion of L4–L6 led to a decrease of about 3000 motor fibers, which was depicted by the axonal counting. A greater number of axons could be seen in the reimplanted groups, combined with the presence of a close to normal myelin sheath. In contrast, avulsion only derived nerves displayed significantly less large dimension axons indicating the loss of motor axons. In line with that, Lang *et al*. (2005) [30] observed that the mean axonal diameter was significantly higher in regenerated axons that left the CNS compartment through the original ventral exit zones, similar to those presented herein, in comparison with axons that regenerated through the lateral funiculus.

Additionally to fiber dimensions, we also analyzed the “g” ratio (RZG), a morphometric parameter that expresses the relationship between Schwann cells and axons during functional nerve regeneration [31,32,33]. Herein we demonstrated that the implanted groups were significantly more similar to the contralateral nerve than the avulsion only rats. This indicates that the fibrin sealant implant provided conditions for regeneration of nerve fibers, resulting in a close to normal “g” ratio distribution. These results are in accordance with already published data by our group, where improvement in motor function was observed at 12 weeks after reimplantation of avulsioned roots [18,27]. MC treatment, however, resulted in decreased RZG, which will be addressed in future short-term analysis. Overall, the present work indicates that reimplantation of ventral roots at the site of injury is neuroprotective and results in preservation of spinal circuits, as seen by the ultrastructural evaluation of synapses.

## 4. Experimental Section

### 4.1. Experimental Animals

Thirty adult female Lewis (LEW/HsdUnib) rats (7 weeks old) were obtained from the multidisciplinary Center for Biological Investigation (CEMIB/UNICAMP) and housed under a 12 h light/dark cycle with free access to food and water. The study was approved by the Institutional Committee for Ethics in Animal Experimentation (Committee for Ethics in Animal Use—Institute of Biology—CEUA/IB/UNICAMP, proc. Nr. 2073-1) on 8 February 2010 (Campinas-SP, Brazil). All experiments were performed in accordance with the guidelines of the Brazilian College for Animal Experimentation. The animals were subjected to unilateral avulsion of the L4–L6 ventral roots, and analyzed at 4 and 12 weeks after injury. The animals were divided into three groups: (1) VRA without reimplantation (avulsion, *n* = 10); (2) VRA followed by lesioned roots reimplantation (avulsion + sealant, *n* = 10) and (3) VRA followed by lesioned roots reimplantation with fibrin sealant and MC added to the sealant (avulsion + sealant + cells, *n* = 10). Four and twelve weeks after injury, the animals were killed and the lumbar spinal cords were processed for transmission electron microscopy and the sciatic nerves were processed for myelinated axon counting and morphometry. The contralateral sciatic nerve and spinal cord antimere were used as controls throughout the study.

### 4.2. VRA

The rats were anesthetized with 50 mg/kg of ketamine (Vetaset^®^, Fort Dodge Animal Health, Fort Dodge, IA, USA) and 10 mg/kg of xylazine (Kensol^®^, Konig SA, Buenos Aires, Argentina) and subjected to unilateral avulsion of the lumbar ventral roots as previously described [27,34,35,36,37,38]. Unilateral avulsion of ventral roots was performed at the L4, L5 and L6 lumbar spinal cord segments, after unilateral laminectomy on the right side. A longitudinal incision was made to open the dural sac, and the denticulate ligament was dissected. Finally, the ventral roots associated with the lumbar intumescence were identified and avulsed with a fine forceps (Dumont^®^, Switzerland, Part number 11242-40). After lesioning, the musculature, fascia and skin were sutured in layers. Chlorhydrate of tramadol was administrated by gavage after the surgical procedures (20 mg/kg) and 2.5 mg/day soluble in water, over 5 days.

### 4.3. Reimplantation of the Motor Roots

In the avulsion + sealant and avulsion + sealant + cells groups, the roots were replaced at the exact point of detachment, on the ventral surface of the lumbar spinal cord at the avulsion site with the aid of a snake venom fibrin sealant [27]. The fibrin sealant used in this study was provided by the Center for the Study of Venoms and Venomous Animals (CEVAP), UNESP, Brazil (patents BR 10 2014 011432 7 and BR 10 2014 011436 0) [39,40,41]. The sealant used herein was composed of three components, namely the cryoprecipitate rich in fibrinogen derived from bubaline blood, calcium chloride solution and thrombin-like enriched solution. During surgical repair of the avulsed roots, the first two components were applied and the avulsed roots were returned to their original sites and, in the avulsion + sealant + cells group, the cells were added at this point. The third component was then added for the sealant polymerization. The stability of the root fixation was observed to evaluate the success of the repair.

### 4.4. Mononuclear Cells (MC) Transplantation

MC were extracted from transgenic Lewis rats (LEW-Tg EGFP F455/Rrrc—The animals were imported from Missouri University (EUA) and were provided by Alfredo Miranda Góes, Federal University of Minas Gerais (UFMG), Minas Gerais, Brazil) carrying the *EGFP* (Enhanced green fluorescent protein) gene. The EGFP rats were killed with a lethal dose of halothane (Tanohalo, Cristália Chemicals and Pharmaceuticals, Itapira-SP, Brazil). Bone marrow from the femur and the tibia was collected and the cells were isolated by gradient density centrifugation using Histopaque^®^ 1077 (Sigma Aldrich, Steinheim, Germany), following the separation of mononuclear cells method from Sigma–Aldrich protocol number 1119 (Sigma Aldrich, Steinheim, Germany). The cell suspension consists of heterogeneous cell populations (13.2% CD11b, 1.52% CD3, 92.2% CD45, 20.8% CD34 of CD45). After two washing steps, cells were immediately transplanted at the avulsed root site. In avulsion + sealant + cells group, 3 × 10^5^ MCs were added to the fibrin sealant in reimplanted avulsed roots at the moment of reimplantation.

### 4.5. Specimen Preparation

At the predetermined times, the animals were killed with a lethal dose of halothane (Tanohalo, Cristália Chemicals and Pharmaceuticals, Itapira-SP, Brazil) and the vascular system was transcardially perfused. For this, phosphate buffer 0.1 M (pH 7.4) was perfused through the left ventricle, followed by fixative containing 2.5% glutaraldehyde and 0.5% paraformaldehyde in the same buffer (pH 7.4). The lumbar spinal cord and a 10 mm mid-thigh segment of the sciatic nerve (ipsi and contralateral), were removed and stored overnight in the same fixative solution, at 4 °C. The specimens were then trimmed and osmicated, dehydrated through ethanol series, and embedded in Durcupan ACM (Fluka, Steinheim, Switzerland). The blocks were trimmed and semi-thin sections (0.5 µm) obtained and stained with 0.25% toluidine blue for light microscopy observation. Ultrathin sections (500 A), from the L4–L6 segments, were made in an ultramicrotome (Leica Ultracut UCT Ultramicrotome), collected on formvar-coated single slot grids, contrasted with uranyl acetate and lead citrate, and examined under a Tecnai G^2^ Spirit BioTwin (FEI, Eindhoven, The Netherlands) transmission electron microscope.

### 4.6. Synaptic Analysis of the Ultrathin Sections

Neurons with large cell bodies (>35 µm in diameter), found in the sciatic motoneuron pool and cut in the nuclear plane, were identified as α motoneurons by the presence of *C*-type nerve terminals. The surfaces of these cells were then sequentially digitized at a magnification of 13,000× using a video camera (Eagle, FEI, Eindhoven, The Netherlands) connected to a computerized system. The images were then mounted together in vectorial software (Adobe Photoshop CS4 extended, Adobe Systems Incorporated, San Jose, CA, USA), and the total perimeter of the neurons was measured. Synaptic terminals apposing the motoneuron soma were identified, and their numbers per 100 µm of cell membrane and length of apposition, as a percentage of membrane length, were calculated using the measurement tool of the ImageTool software (UTHSCSA, San Antonio, TX, USA). The terminals were typed under high magnification as *F* (with flattened synaptic vesicles, inhibitory inputs), *S* (with spherical synaptic vesicles, excitatory glutamatergic inputs), or *C* (cholinergic inputs), according to the nomenclature of Conradi [42]. The distance between consecutive nerve terminals covering the motoneurons was also determined. In total, two neurons per animal in each group of five animals were examined (*n* = 10 neurons for each group). The data are presented as mean ± SE.

### 4.7. Sciatic Nerve Regeneration

The sciatic nerves were processed for myelinated axon counting and morphometric evaluation by transverse semithin sections (0.5 μm thick), stained in toluidine blue solution, and examined under light microscopy. The analyses were performed by sampling at least 30% of each nerve cross-section (magnification of 1000×), generating approximately 15 images per animal. The images were used for counting the total number of myelinated axons in each specimen. Also, two fields were used to measure the diameter of myelinated fibers and the myelinated axon (Adobe Photoshop CS4 extended, Adobe Systems Incorporated, San Jose, CA, USA). These values were used to calculate ‘‘g’’ ratio (axon diameter/fiber diameter).

### 4.8. Statistical Analysis

Ultrastructural quantification data and counting of myelinated fibers from sciatic nerve were firstly evaluated with one-way ANOVA. Data from morphometry of myelinated fibers from the sciatic nerve were evaluated with two-way ANOVA. In both cases the Bonferroni post-test was used to identify intergroup differences.

## 5. Conclusions

Ventral root reimplantation after avulsion, at the site of injury, is neuroprotective and results in preservation of spinal circuits, seen by the ultrastructural evaluation of synapses. Mononuclear cell administration, associated with fibrin sealant reimplantation did not provide further regenerative improvements, possibly due to the long-term evaluation of the present work (12 weeks). Importantly, the results herein provide a basis for future use of fibrin sealant derived from snake venom to repair proximal CNS/PNS lesions.

## References

[1] Thatte M.R., Babhulkar S., Hiremath A. (2013). Brachial plexus injury in adults: Diagnosis and surgical treatment strategies. Ann. Indian Acad. Neurol..

[2] Carlstedt T. (2009). Nerve root replantation. Neurosurg. Clin. N. Am..

[3] Koliatsos V.E., Price W.L., Pardo C.A., Price D.L. (1994). Ventral root avulsion: An experimental model of death of adult motor neurons. J. Comp. Neurol..

[4] Chu T.H., Wu W. (2009). Neurotrophic factor treatment after spinal root avulsion injury. Cent. Nerv. Syst. Agents Med. Chem..

[5] Brannstrom T., Kellerth J.O. (1998). Changes in synaptology of adult cat spinal α-motoneurons after axotomy. Exp. Brain Res..

[6] Cullheim S., Carlstedt T., Risling M. (1999). Axon regeneration of spinal motoneurons following a lesion at the cord-ventral root interface. Spinal Cord.

[7] Hoang T.X., Nieto J.H., Dobkin B.H., Tillakaratne N.J., Havton L.A. (2006). Acute implantation of an avulsed lumbosacral ventral root into the rat conus medullaris promotes neuroprotection and graft reinnervation by autonomic and motor neurons. Neuroscience.

[8] Cullheim S., Carlstedt T., Linda H., Risling M., Ulfhake B. (1989). Motoneurons reinnervate skeletal muscle after ventral root implantation into the spinal cord of the cat. Neuroscience.

[9] Chang H.Y., Havton L.A. (2008). Surgical implantation of avulsed lumbosacral ventral roots promotes restoration of bladder morphology in rats. Exp. Neurol..

[10] Hallin R.G., Carlstedt T., Nilsson-Remahl I., Risling M. (1999). Spinal cord implantation of avulsed ventral roots in primates: Correlation between restored motor function and morphology. Exp. Brain Res..

[11] Risling M., Linda H., Cullheim S., Franson P. (1989). A persistent defect in the blood-brain barrier after ventral funiculus lesion in adult cats: Implications for CNS regeneration?. Brain Res..

[12] Sjogren A.M., Thelestam M., Blomqvist L., Linda H., Remahl S., Risling M. (1991). Extravasation of staphylococcal α-toxin in normal and injured CNS regions lacking blood-brain barrier function: Observations after ventral root replantation. Brain Res..

[13] Frisen J., Fried K., Sjogren A.M., Risling M. (1993). Growth of ascending spinal axons in CNS scar tissue. Int. J. Dev. Neurosci..

[14] Lopes-Filho J.D., Caldas H.C., Santos F.C., Mazzer N., Simoes G.F., Kawasaki-Oyama R.S., Abbud-Filho M., Oliveira A.R., Toboga S.R., Chueire A.G. (2011). Microscopic evidences that bone marrow mononuclear cell treatment improves sciatic nerve regeneration after neurorrhaphy. Microsc. Res. Tech..

[15] Kachramanoglou C., Li D., Andrews P., East C., Carlstedt T., Raisman G., Choi D. (2011). Novel strategies in brachial plexus repair after traumatic avulsion. Br. J. Neurosurg..

[16] Spejo A.B., Carvalho J.L., Goes A.M., Oliveira A.L. (2013). Neuroprotective effects of mesenchymal stem cells on spinal motoneurons following ventral root axotomy: Synapse stability and axonal regeneration. Neuroscience.

[17] Li Y., Yamamoto M., Raisman G., Choi D., Carlstedt T. (2007). An experimental model of ventral root repair showing the beneficial effect of transplanting olfactory ensheathing cells. Neurosurgery.

[18] Barbizan R., Castro M.V., Barraviera B., Ferreira R.S., Oliveira A.L. (2014). Influence of delivery method on neuroprotection by bone marrow mononuclear cell therapy following ventral root reimplantation with fibrin sealant. PLoS One.

[19] Cullheim S., Wallquist W., Hammarberg H., Linda H., Piehl F., Carlstedt T., Risling M. (2002). Properties of motoneurons underlying their regenerative capacity after axon lesions in the ventral funiculus or at the surface of the spinal cord. Brain Res. Rev..

[20] Hammarberg H., Piehl F., Risling M., Cullheim S. (2000). Differential regulation of trophic factor receptor mRNAs in spinal motoneurons after sciatic nerve transection and ventral root avulsion in the rat. J. Comp. Neurol..

[21] Carlstedt T., Risling M., Linda H., Cullheim S., Ulfhake B., Sjogren A.M. (1990). Regeneration after spinal nerve root injury. Restor. Neurol. Neurosci..

[22] Carlstedt T., Linda H., Cullheim S., Risling M. (1986). Reinnervation of hind limb muscles after ventral root avulsion and implantation in the lumbar spinal cord of the adult rat. Acta Physiol. Scand..

[23] Purves D., Lichtman J.W. (1978). Formation and maintenance of synaptic connections in autonomic ganglia. Physiol. Rev..

[24] Purves D. (1975). Functional and structural changes in mammalian sympathetic neurones following interruption of their axons. J. Physiol..

[25] Takata M., Nagahama T. (1983). Synaptic efficacy of inhibitory synapses in hypoglossal motoneurons after transection of the hypoglossal nerves. Neuroscience.

[26] Delgadogarcia J.M., Delpozo F., Spencer R.F., Baker R. (1988). Behavior of neurons in the abducens nucleus of the alert cat III. Axotomized motoneurons. Neuroscience.

[27] Barbizan R., Castro M.V., Rodrigues A.C., Barraviera B., Ferreira R.S., Oliveira A.L. (2013). Motor recovery and synaptic preservation after ventral root avulsion and repair with a fibrin sealant derived from snake venom. PLoS One.

[28] Linda H., Shupliakov O., Ornung G., Ottersen O.P., Storm-Mathisen J., Risling M., Cullheim S. (2000). Ultrastructural evidence for a preferential elimination of glutamate-immunoreactive synaptic terminals from spinal motoneurons after intramedullary axotomy. J. Comp. Neurol..

[29] Linda H., Risling M., Cullheim S. (1985). “Dendraxons” in regenerating motoneurons in the cat: Do dendrites generate new axons after central axotomy?. Brain Res..

[30] Lang E.M., Asan E., Plesnila N., Hofmann G.O., Sendtner M. (2005). Motoneuron survival after C7 nerve root avulsion and replantation in the adult rabbit: Effects of local ciliary neurotrophic factor and brain-derived neurotrophic factor application. Plastic Reconstr. Surg..

[31] Ikeda M., Oka Y. (2012). The relationship between nerve conduction velocity and fiber morphology during peripheral nerve regeneration. Brain Behav..

[32] Waxman S.G. (1980). Determinants of conduction velocity in myelinated nerve fibers. Muscle Nerve.

[33] Waxman S.G. (1980). Pathophysiology of nerve conduction: Relation to diabetic neuropathy. Ann. Intern. Med..

[34] De Freria C.M., Barbizan R., de Oliveira A.L. (2012). Granulocyte colony stimulating factor neuroprotective effects on spinal motoneurons after ventral root avulsion. Synapse.

[35] Barbizan R., Oliveira A.L. (2010). Impact of acute inflammation on spinal motoneuron synaptic plasticity following ventral root avulsion. J. Neuroinflam..

[36] Scorisa J.M., Zanon R.G., Freria C.M., de Oliveira A.L. (2009). Glatiramer acetate positively influences spinal motoneuron survival and synaptic plasticity after ventral root avulsion. Neurosci. Lett..

[37] Rodrigues Hell R.C., Silva Costa M.M., Goes A.M., Oliveira A.L. (2009). Local injection of BDNF producing mesenchymal stem cells increases neuronal survival and synaptic stability following ventral root avulsion. Neurobiol. Dis..

[38] Oliveira A.L., Langone F. (2000). GM-1 ganglioside treatment reduces motoneuron death after ventral root avulsion in adult rats. Neurosci. Lett..

[39] Barros L.C., Soares A.M., Costa F.L., Rodrigues V.M., Fuly A.L., Giglio J.R., Gallacci M., Thomazini-Santos I.A., Barraviera S.R.C.S., Barraviera B. (2011). Biochemical and biological evaluation of gyroxin isolated from *Crotalus durissus* terrificus venom. J. Venom. Anim. Toxins Incl. Trop. Dis..

[40] Barros L.C., Ferreira R.S., Barraviera S.R., Stolf H.O., Thomazini-Santos I.A., Mendes-Giannini M.J., Toscano E., Barraviera B. (2009). A new fibrin sealant from *Crotalus durissus* terrificus venom: Applications in medicine. J. Toxicol. Environ. Health Part B Crit. Rev..

[41] Gasparotto V.P., Landim-Alvarenga F.C., Oliveira A.L., Simoes G.F., Lima-Neto J.F., Barraviera B., Ferreira R.S. (2014). A new fibrin sealant as a three-dimensional scaffold candidate for mesenchymal stem cells. Stem Cell Res. Ther..

[42] Conradi S. (1969). Observations on the ultrastructure of the axon hillock and initial axon segment of lumbosacral motoneurons in the cat. Acta Physiol. Scand. Suppl..

